# Evaluation of physicochemical properties and volatile compounds of Chinese dried pork loin curing with plasma-treated water brine

**DOI:** 10.1038/s41598-019-50351-5

**Published:** 2019-09-24

**Authors:** Ji Luo, Wenjing Yan, Mustapha Muhammad Nasiru, Hong Zhuang, Guanghong Zhou, Jianhao Zhang

**Affiliations:** 1 National Center of Meat Quality and Safety Control, Synergetic Innovation Center of Food Safety and Nutrition, College of Food Science and Technology, Nanjing Agricultural University, Nanjing, Jiangsu 210095 PR China; 2 Quality and Safety Assessment Research Unit, 950 College Station Road, Athens, GA 30605 United States

**Keywords:** Characterization and analytical techniques, Mass spectrometry, Laser-produced plasmas, Chemical physics, Process chemistry

## Abstract

The application of dielectric barrier discharge cold plasma (DBD-CP)-treated water as a novel curing process for manufacturing Chinese dried pork loin was investigated. The treatment time of DBD-CP was optimized based on the pH and nitrite level of the plasma-treated water (PTW). PTW treated for 3 min had an alkaline environment and a higher nitrite content than that at the other lengths of treatment time. Pork loins were marinated in control or PTW brine at 4 °C for 24 h and then dry-ripened for 15 days. PTW with a higher treatment voltage significantly decreased lipid oxidation of the products and led to an increased a* value (redness) and an increased residual nitrite content in products that was still within the range of use (all *P* < 0.05). The contents of each free amino acid increased with increasing treatment intensity (*P* < 0.05). There were 22 new volatile compounds generated in PTW-cured products, such as 3-methyl-butanol, hexanal and 2,3-octanedione, while six substances were lost, such as 2-pentylfuran, compared with those in the control. This study indicates that using PTW as a curing method can be a potential and effective way of producing dried pork meat products.

## Introduction

Dielectric barrier discharge cold plasma (DBD-CP) is an emerging technology that has been widely applied for the functionalization and modification of proteins^1,2^, the elimination of pathogenic and spoilage microorganisms^3,4^ and the curing process^5,6^ of meat products. DBD-CP was used for killing microorganisms in fresh pork loin and extending the shelf life of fresh meat^7^. Lee *et al*.^5^ reported that direct DBD-CP treatment can be used as a curing process to produce canned ground ham without undesirable sensory properties due to the generation of nitrite in the meat batter after DBD-CP treatment. For the meat batter, the utilization of direct DBD-CP treatment could increase the a* value and generate nitrites in meat batter without sterilization effects^8^. Hae *et al*.^9^ treated marinated pork with DBD-CP and found that the use of DBD-CP had a bactericidal effect on pork jerky and could improve the formation of a cured color in pork jerk processing. Jung *et al*.^6^ processed sausage with DBD-treated water, celery powder and synthetic sodium nitrite and found that PTW could be used as a nitrite source for curing emulsion-type sausage, and PTW-treated sausages had similar sensory properties to sodium nitrite-treated sausages. In addition, PTW could also be applied in manufacturing loin ham as a replacement for sodium nitrite. The residual nitrite content after PTW treatment was significantly lower than that of the sodium nitrite treatment, and the loin ham produced by PTW was proven to not be genotoxic by the Ames test^10^.

In this study, PTW was formed by dielectric barrier discharge cold plasma (DBD-CP) treatment of water. PTW, which means water containing nitrite^11^, contains many reactive oxygen and nitrogen species (ROS and RNS, respectively)^12^. Nitrite has the ability to develop a red color, inhibit lipid oxidation, inactivate spoilage and pathogenic microorganisms, and form a unique flavor in meat products^6^; thus, PTW has the potential to be a nitrite source for cured meat products.

Currently, consumers are increasingly demanding “chemical-free” meat products. Celery powder has been used in cured meat products to replace synthetic nitrite because of its high nitrate content^5,6^. Compared with celery powder, PTW is a more welcome alternative due to its low price and short processing time; moreover, the use of celery powder in meat processing could result in an undesirable aroma^6^.

There are already plenty of meats and meat products processed by DBD-CP, such as canned ground ham^5^, emulsion-type sausage^6^, meat batter^8^, pork jerky^9^ and loin ham^10^. Meat can be treated by injecting PTW into the meat or by direct treatment with DBD-CP. However, studies on the volatile and nonvolatile compounds of meat products cured with DBD-CP are very limited, and these compounds can have a great impact on the acceptability of these products by consumers.

To the best of our knowledge, the use of PTW as a curing process has not been evaluated by analysis of the physicochemical properties and flavor of dried pork loin. Furthermore, an increasing number of meat factories use frozen meat as raw material^13^, and the application of PTW as a simultaneous thawing/curing method would lower the thawing/curing time, reduce the cost and control the quality of the product. The objective of this research was to explore changes in the quality characteristics, lipid oxidation, free amino acid contents and volatile compounds of dried pork loin cured with PTW. The findings of this study will be very useful for the development of meat products in the future.

## Materials and Methods

### Preparation of PTW

**Figure 1:**
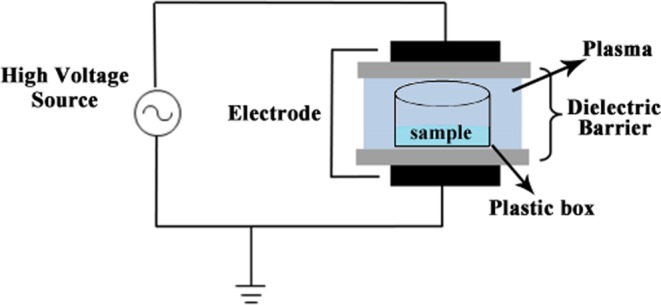
Schematic diagram of dielectric barrier discharges cold plasma (DBD-CP) system. The plasma discharge was generated under the following conditions: operating frequency, 50 kHz; the output voltages were 50, 60 and 70 kV; the current were 0.7, 0.8 and 0.9 mA, respectively, the power were 35, 48 and 63 w, respectively. All the treatments were carried out using ambient air without air circulation or supplementation with any other particular gas at an atmospheric pressure environment. The distance between the ground electrode and the water sample was adjusted to 2 mm, the gap between water surface and the top electrode was fixed at 10 mm during DBD-CP treatments.

As demonstrated in Fig. 1, DBD-CP treatment was carried out using the same system as described by Huang *et al*.^7^. In our DBD system, the output voltage was determined by a high-voltage probe (Tektronix Inc., P6015A, USA) and a digital oscilloscope (Tektronix Inc., 2024C, USA). The discharge current was implemented using a conventional current monitor (Pearson Electronics, 2877, USA). The peak output power (P) was calculated by the following formula: P = V × I, where V is the output voltage and I is the discharge current. When the output voltage was 50, 60 and 70 kV, the current was 0.7, 0.8 and 0.9 mA, respectively, so the power was 35, 48 and 63 W, respectively. The operating frequency of our DBD system was 50 Hz. All treatments were carried out using ambient air without air circulation or supplementation with any other particular gas in an atmospheric pressure environment. The distance between the ground electrode and the water sample was adjusted to 2 mm, and the gap between the water surface and the top electrode was fixed at 10 mm during DBD-CP treatments. Distilled water with 0.5% sodium pyrophosphate (w/v) was added to a polypropylene box (178 × 126 × 35 mm), which was then sealed with a laminated barrier film made of polyamide/polyethylene. The box with distilled water was put in the center of two cyclic aluminum annular electrodes (150 mm diameter) between two polypropylene layers above and below the box, which were used as dielectric barriers and were placed at a distance of 40 mm. Afterwards, the box was subjected to DBD-CP treatment at 50, 60, and 70 kV, representing low power, medium power, and high power, respectively. The treatment voltage in this study was used according to the Paschen curves for air breakdown, our DBD-CP system and previously published studies^14–16^. Our preliminary experiments found that there was no significant difference in physicochemical properties between the treated and untreated products when the treatment voltage was below 50 kV. In addition, when the treatment voltage exceeded 75 kV, it resulted in frequent electric arcing with our DBD-CP system and induced excessive protein oxidation, which could lead to bad flavor and adverse effects on meat quality, so we selected 50, 60, and 70 kV as the treatment voltages.

Each level of plasma treatment intensity was performed for 1, 2, 3, 4, 5 and 6 min. To ensure the consistency of liquid samples and prevent the interference of the detection reactions with dissolved salt components, PTW was produced using distilled water containing 0.5% sodium pyrophosphate (w/v) under various DBD-CP treatment times and voltages. After each treatment, sodium chloride (NaCl) was added to the PTW to prepare PTW brine solutions, and the concentration of NaCl in all PTW brine solutions was maintained at 6%. Sodium pyrophosphate was used to maintain an alkaline environment. All treatments were conducted under 40 ± 5% relative humidity (RH) and 20 ± 2 °C. At the end of all treatments, the PTW temperature did not exceed 35 °C. Each treatment was carried out in triplicate.

### Physiochemical properties of PTW

As soon as each DBD-CP treatment was completed, 50 mL of PTW was collected for analysis of nitrite content and pH. A pH meter (Testo 230, Testo AG Lenzkirch, Germany) was used to determine the pH of the PTW brine. The nitrite content in PTW was measured by spectrophotometry according to Tian *et al*.^17^. After each DBD-CP treatment, 10 mL of PTW was added to 100 mL of 10 g/L sulfanilamide at room temperature for 2 min and then mixed with 100 mL of 1.0 g/L N-(1-naphthyl)-ethylenediamine hydrochloride for 20 min. The concentration of nitrite was determined by measuring the absorbance at 540 nm. The diazotizing reagent and coupling reagent were sulfanilamide and N-(1-naphthyl)-ethylenediamine hydrochloride, respectively.

### Manufacturing and sample collection of chinese dried pork loins

**Figure 2:**
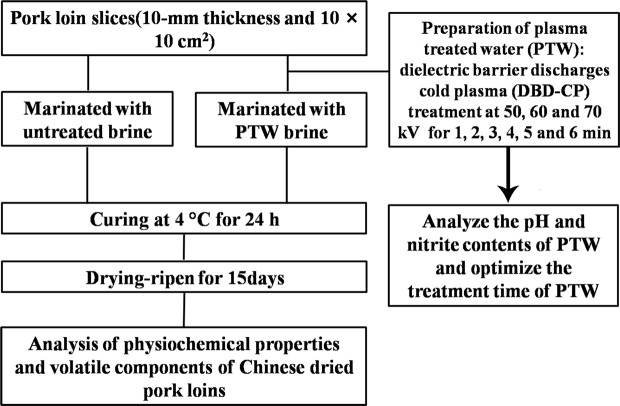
Experimental design and process of Chinese dried pork loin production.

Pork loins (lumbar longissimus muscle) of Taihu black pigs were obtained from a local market (Nanjing city, Jiangsu Province, China). Taihu black pigs are rich in lean meat with a fresh meat flavor. Pork loins were sliced to a 10-mm thickness and a size of 10 × 10 cm^2^, randomly divided into four groups, and subsequently vacuum packed and refrigerated at 4 °C before use. One group of samples was prepared as a control (n = 10), and the others used PTW brine as the curing process (n = 100). Figure 2 shows the experimental flow chart. At first, each pork loin was marinated at 4 °C for 24 h. After marinating, the samples were removed from the treatment tank and dry-ripened for 15 days. According to our preliminary experiment^18^ with slight modification, the temperature was gradually increased from 12 °C to 37 °C at 2 °C per day; simultaneously, the RH progressively decreased from 80% to 65% at 2% per day during the ripening period. For physiochemical properties and volatile analysis, three ripened dried pork loin samples (approximately 50 g each) were randomly taken from each treatment, and all samples were vacuum-packed and stored at −40 °C.

### pH measurement

Samples of ripened dried pork loin (5 g) were homogenized with 5 volumes of distilled water at 10,000 rpm for 1 min using a homogenizer model T18 (IKA- Werke GmbH& Co., Staufen, KG, Germany). A pH meter (Testo 230, Testo AG Lenzkirch, Germany) was used to measure the pH value of the homogenates.

### Color measurement

CIE L* (lightness), a* (redness) and b* (yellowness) values of dried pork loin were assessed using a colorimeter (Model CR-400; Konica Minolta, Osaka, Japan) with a 30 mm diameter measurement area, an Illuminant D65, and a 0° observer angle. The surface sample color was measured perpendicularly at three different sites. The instrument was calibrated to standard white and black tiles before analysis.

### Determination of nitrosoheme pigment and nitrite concentration

Nitrosoheme pigment extraction and determination were performed by method published in previous paper^19^. Acetone solution (80%) was prepared to extract the pigments in dried pork loins; the solutions were then filtered through Whatman 40 filter papers (Whatman PLC., Maidstone, UK). The concentration of nitrosoheme pigment (ppm of hematin) was calculated as A540 × 290 after measuring the absorbance at 540 nm with a spectrophotometer (UV-2450, Shimadzu Co., Kyoto, Japan). Residual nitrite content in dried pork loin was determined based on the Association of Official Analytical Chemists method NO. 973.31^20^.

### Determination of 2-thiobarbituric acid reactive substances (TBARS)

The extent of lipid oxidation, as measured by the level of 2-thiobarbituric acid reactive substances (TBARS), was determined according to a previously published method with some modifications^21^. Ten grams of dried pork loin samples was homogenized with 30 mL of a 25% trichloroacetic acid (TCA) solution containing 2% TBA at 10,000 rpm for 1 min and then filtered. Ten milliliters of 20 mM TCA was mixed with 10 mL filtrate for 2 min. The solution was heated in a water bath at 80 °C for 30 min and then cooled with flowing water for 10 min before centrifugation at 10,000 × *g* for 5 min (4 °C). The absorbance of supernatants was recorded at 532 nm. The determination of malondialdehyde (MDA) was based on a standard curve constructed using 1,1,3,3-tetraethoxypropane. The TBARS value was expressed as mg MDA/kg of sample.

### Free amino acid analysis

The free amino acids (FAAs) were extracted from the *biceps femoris* muscle of dried pork loin according to the method described by Lorenzo *et al*.^22^ with some modifications. In general, 2 g of each sample was homogenized with 25 mL of TCA and incubated for 1 h before 5 mL of the solution was filtered through a 0.45-μm membrane. Afterwards, the filtrate was identified and quantified by an automated amino acid analyzer (L-8900; Hitachi, Tokyo, Japan). FAAs were determined for each sample in triplicate. The concentration of FAAs was expressed as mg/100 g of dry matter.

### Volatile compound analysis

The determination of the volatile compounds in the pork loin samples was performed using a previously published method with slight modifications^23^. Samples of 5.0 ± 0.3 g were put into a 20 mL headspace sample vial and sealed with a PTFE-faced silicone rubber septum. The vial was equilibrated at 40 °C in a water bath. Solid-phase microextraction (SPME) and gas chromatography-mass spectrometry (GC/MS) (Agilent 7890) analysis were applied to determine the headspace aroma compounds. After incubating, a 75 μm Carboxen/poly (dimethylsiloxane) (CAR/PDMS) fiber (Supelco, Bellefonte, PA, USA) was inserted into the vial to absorb the aroma compounds at 30 °C for 30 min. Then, the fiber was injected into the GC to desorb at 220 °C for 5 min in splitless mode. The temperature of the GC oven began at 35 °C for 3 min and then was raised to 80 °C at 5 °C/min; the oven temperature was then raised at 8 °C/min to 150 °C, and finally, the temperature was raised to 220 °C at 10 °C/min and held for 10 min. The detector temperature was set at 240 °C. The volatile compounds were separated on a DB5MS capillary column (60 m × 0.32 mm × 1.8 μm film thickness) (Sigma, USA) using helium as the carrier gas. The mass spectra were obtained using a mass selective detector after ionization by electronic impact ionization (70 eV). The detector voltage was 350 V with a scan range between 30 and 450 m/z at a rate of 6.34 scans/s over the range. Data were expressed as the percentage of the total area of identified peaks and identified by comparing their mass spectra with those in the databases of NISTDEMO and the WILEY library.

### Statistical analysis

Three samples were randomly taken from ripened Chinese dried pork loin for each treatment, and each sample was analyzed in triplicate for individual parameters. All data were analyzed by one-way analysis of variance (ANOVA) using SPSS version 19.0 (SPSS Inc., Chicago, USA). Significant differences (*P* < 0.05) among means were identified by Duncan’s new multiple range tests.

## Results and Discussion

### Physiochemical properties of PTW

**Figure 3:**
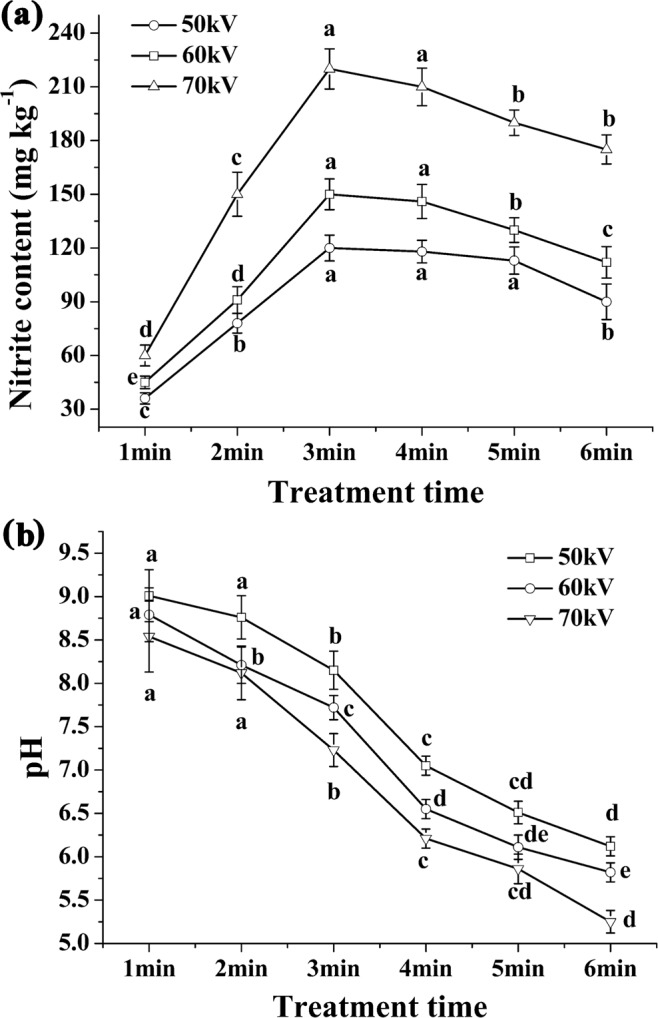
Nitrite content (mg kg^−1^) and pH of the PTW brine treated with DBD-CP at 50, 60 and 70 kV, respectively. PTW, plasma treated water. Different letters among PTW treated with DBD-CP for different time periods (1, 2, 3, 4, 5, and 6 min) differ significantly (*P* < 0.05).

The nitrite concentration and pH value of PTW were analyzed as soon as each DBD-CP treatment was completed. The physiochemical properties of the PTW brine treated with different voltage intensities and time are shown in Fig. 3. Clearly, after treatment at all the intensities, the nitrite level of the brine increased significantly up to 3 min of treatment and then began to decline with increasing treatment time, which was similar to the previous results^12,24,25^. We speculated that the rise in the nitrite level during the first 3 min of treatment was attributed to the nitrogen oxides generated by ionization of atmospheric oxygen and nitrogen molecules^5^, such as NO, NO_2_, N_2_O_4_, and N_2_O_3_, which are known to form nitric and nitrous acids after reacting with H_2_O^12^:

1 $${\rm{NO}}+{{\rm{NO}}}_{2}+{{\rm{H}}}_{2}{\rm{O}}\to 2{{\rm{HNO}}}_{2}$$

2 $$2{{\rm{NO}}}_{2}+{{\rm{H}}}_{2}O\to {{\rm{HNO}}}_{2}+{{\rm{HNO}}}_{3}$$

3 $${{\rm{N}}}_{2}{{\rm{O}}}_{4}+{{\rm{H}}}_{2}{\rm{O}}\to {{\rm{HNO}}}_{2}+{{\rm{HNO}}}_{3}$$

4 $${{\rm{N}}}_{2}{{\rm{O}}}_{3}+{{\rm{H}}}_{2}{\rm{O}}\to 2{{\rm{HNO}}}_{2}$$

5 $$4{\rm{NO}}+{{\rm{O}}}_{2}+2{{\rm{H}}}_{2}{\rm{O}}\to 4{{\rm{HNO}}}_{2}$$

With a similar DBD system^6^, Ercan *et al*.^24^ have reported that the nitrite concentration in the liquid increased after plasma treatment.

The reduced nitrite level from 3 to 6 min of treatment could be explained by the decrease in pH. As the pH dropped below 7, nitrite could be decomposed into nitrate and NO (Reaction 6)^26^. After DBD-CP treatment, the decrease in pH could also be related to H_2_O dissociation, thus increasing the H^+^ concentration^27^. In addition, H_2_O_2_ generated in air or liquid after treatment could lead to the formation of H_3_O^+^ ions in PTW^2^.

6 $${{\rm{H}}}_{2}{\rm{O}}+{\rm{e}}\to {{\rm{H}}}^{+}+{\rm{OH}}\,\cdot +2{\rm{e}}-$$

7 $$2{\rm{NO}}+{{\rm{O}}}_{2}\to 2{{\rm{NO}}}_{2};\,{{\rm{NO}}}_{2}+{\rm{OH}}\to {{\rm{HNO}}}_{3}$$

8 $$3{{\rm{NO}}}_{2}^{-}+3{{\rm{H}}}^{+}+{{\rm{H}}}_{2}{\rm{O}}\to 2{\rm{NO}}+{{\rm{NO}}}_{3}^{-}+2{{\rm{H}}}_{3}{{\rm{O}}}^{+12}$$

Due to the strong oxidative characteristic of PTW, which is due to the generation of ROS^12^, NO can be irreversibly oxidized to NO_2_, resulting in the decline of nitrous acid and nitrite contents over time.

9 $$3{{\rm{HNO}}}_{2}\leftrightarrow {{\rm{HNO}}}_{3}+2{\rm{NO}}+{{\rm{H}}}_{2}{\rm{O}}$$

As reported^28^, the decomposition of nitrite generated by plasma treatment would be inhibited under alkaline conditions. Hence, we added sodium pyrophosphate into distilled water to create an alkaline environment. The treatment time of PTW was optimized based on the pH and nitrite contents of PTW. PTW with 3 min of treatment had the most nitrite content and could maintain an alkaline environment at each treatment voltage.

### Color and nitrosoheme pigment

The L* values of dried pork loins were not influenced by PTW treatment (*P* > 0.05, Table 1), which agreed with the previous results^4,29^.

**Table 1:** Instrumental color (L*, a*, and b*) and nitrosoheme pigment content (ppm of hematin) of dried pork loin cured with PTW brine and the untreated brine.

Treatment	L*	a*	b*	Nitrosoheme pigment (ppm of hematin)
Control	51.63 ± 1.16	10.28 ± 0.26^d^	11.12 ± 0.10^c^	14.85 ± 0.35^d^
50 kV	51.37 ± 1.01	14.21 ± 0.33^c^	11.74 ± 0.13^b^	20.57 ± 0.55^c^
60 kV	50.65 ± 0.92	15.65 ± 0.36^b^	12.29 ± 0.50^a^	23.69 ± 0.72^b^
70 kV	49.86 ± 0.44	17.67 ± 0.41^a^	12.75 ± 0.25^a^	30.78 ± 1.91^a^

PTW, plasma treated water.

Different letters within the same column indicate significant differences (*P* < 0.05).

As shown in Table 1, with the treatment voltages of 50, 60, and 70 kV, the a* values of the products cured with PTW brine increased by 28%, 34% and 42%, respectively, compared with those of the control. We speculated that marination in PTW brine caused the infusion of nitrite into the pork loin, which can significantly increase the a* values of meat products compared with the control^30^. Honikel^28^ reported that the redness of cured meat products was attributed to nitrosoheme pigments generated by the reaction of myoglobin and nitric oxide:

10 $${\rm{NO}}+{\rm{myoglobin}}\to {\rm{NO}}-{\rm{myoglobin}}$$

The nitric oxide came from the decomposition of nitrite. During high-temperature ripening, nitrosomyoglobin degraded into nitrosylmyochromogen, which could maintain a red color^31^. Pork loin cured with PTW had enough nitrite to induce the reaction between myoglobin and NO.

Brewer^32^ found that the increased b* values could be related to the formation of metmyoglobin in the meat. We hypothesized that nitrite generated during plasma treatment could oxidize myoglobin into metmyoglobin, which could lead to the promotion of yellowness in color^7^.

### Residual nitrite content and pH

Compared with the control, the residual nitrite contents in all products processed with PTW brine were significantly increased (*P* < 0.05) (Table 2). The results demonstrated that the nitrite content of dried pork loins was significantly increased during the treatments from 50 to 70 kV (*P* < 0.05). Similarly, Jung *et al*.^8^ reported that an increased plasma treatment intensity led to an increased nitrite content in meat samples. Importantly, the nitrite concentration in each product treated with PTW brine was below 20 mg/kg, which is lower than the maximum allowable content of residual nitrite in meat products of 100 mg/kg^28^.

**Table 2:** Physiochemical properties of dried pork loin cured with PTW brine and the untreated brine.

Physiochemical properties	Control	PTW Treatment		
		50 kV	60 kV	70 kV
Residual nitrite (mg kg^−1^)	0.22 ± 0.05^d^	8.12 ± 0.72^c^	10.11 ± 0.54^b^	15.24 ± 1.10^a^
pH	6.28 ± 0.08^a^	5.92 ± 0.08^b^	5.87 ± 0.04^b^	5.75 ± 0.04^c^
TBARS values (mg MDA kg^−1^)	0.41 ± 0.05^a^	0.33 ± 0.04^b^	0.25 ± 0.02^c^	0.22 ± 0.02^c^

Different letters within the same line indicate significant differences (*P* < 0.05).

TBARS, 2-thiobarbituric acid reactive substances.

PTW, plasma treated water.

The pH of the dried pork loins made with untreated brine and PTW brine at 50, 60 and 70 kV were 6.28, 5.92, 5.87 and 5.75, respectively. As reported^3^, the pH of pork loins dropped significantly after DBD-CP treatment because of the NOx molecules produced by DBD-CP and the increased residual nitrite level in the final products.

### Lipid oxidation

The TBARS values of dried pork loin processed with different curing methods are shown in Table 2. Generally, reactive species such as RNS and ROS generated during DBD-CP treatment can induce lipid oxidation^33^. However, in our research, the TBARS value of the dried pork loins cured with PTW brine was significantly lower (*P* < 0.05) than that of the control. The TBARS values of products cured with PTW brine at 50, 60 and 70 kV were 0.33, 0.25 and 0.22, respectively. Lee *et al*.^5^ and Jung *et al*.^8^ found that meat batters treated with DBD-CP did not exhibit increased lipid oxidation. Previous studies have demonstrated that nitrite addition could inhibit lipid oxidation in meat products^30,34^. Nitrite can react with the iron ions of myoglobin to form nitrosylmyoglobin, resulting in the reduction of free iron, which is an accelerator of lipid oxidation, thus preventing lipid oxidation^6^. Additionally, previous studies revealed that DBD-CP treatment of water could produce hydrogen peroxide, which could react with free iron ions in meat products to form hydroxyl radicals, resulting in the loss of iron ions^24,25^. The TBARS value of each treatment was below 0.5 mg MDA/kg, which indicated that the lipid oxidation of the products may not influence the oxidized flavor between PTW-treated loin and the control based on a study by Greene^35^, who found that the oxidized flavor of meat products would not change with TBARS values under 1.0 mg MDA/kg.

### Free amino acid analysis

Table 3 shows the FAA content of dried pork loins under different treatments. Compared with those in the control, the content of each FAA significantly decreased (*P* < 0.05) after 50 kV treatment and increased in the 60 kV and 70 kV treatments with increasing treatment voltage, which indicated that the 60 kV and 70 kV treatments may accelerate some protein degradation into free amino acids and have no negative effect on the flavor of the products. The main FFAs were alanine, glutamic acid, proline, histidine, lysine, and glycine. Some FAAs have a significant relationship with taste^36^, and some of them are the precursors of flavor compounds for dry-cured meat products^37^. For instance, a sweet flavor is related to the contents of alanine, serine, proline, threonine, and glycine; a bitter flavor is attributed to leucine, valine, isoleucine, and methionine; phenylalanine, histidine, glutamic and aspartic acid are helpful for forming acidic tastes; lysine, tyrosine, and aspartic acid are connected with an aged flavor. Therefore, the accumulation of FAAs has a great influence on the sensory quality of dried pork loins. The arginine content increased with increasing treatment intensity, and its concentration was significantly higher (*P* < 0.05) in all PTW treatments than that in the control. It has been proven that, as one of the umami-related amino acids, arginine is important for developing the characteristic flavor of dry-cured meat products^38^. The cysteine concentration of the control was significantly lower (*P* < 0.05) than that in the 60 kV and 70 kV treatments, which could be related to the higher TBARS values of the control since cysteine was particularly sensitive to oxidative treatment^39^. Compared with the other FAAs, the relatively low concentration of cysteine was due to its involvement in the Maillard reaction^40^ and the dehydrogenation of the thiol group in the cysteine branched chain^7^. Therefore, the low amount of cysteine seen in the 50 kV treatment may be related to the low content of 3-methyl-butanal in the 50 kV treatment that underwent the Maillard reaction.

**Table 3:** Free amino acids concentration (mg/100 g dry matter) in dried pork loin cured with PTW brine and the untreated brine.

FAAs	Control	PTW Treatment		
		50 kV	60 kV	70 kV
**Sweet flavor**				
Ala	185.66 ± 4.02^c^	155.27 ± 2.65^d^	354.14 ± 3.14^b^	426.29 ± 7.21^a^
Thr	43.46 ± 1.25^c^	26.11 ± 0.25^d^	140.51 ± 1.16^a^	121.64 ± 2.18^b^
Gly	22.56 ± 0.21^d^	37.75 ± 0.27^c^	109.46 ± 1.01^a^	90.19 ± 2.03^b^
Pro	174.66 ± 2.36^c^	115.39 ± 1.09^d^	210.28 ± 1.08^b^	264.45 ± 1.17^a^
Ser	18.02 ± 0.25^c^	28.64 ± 0.11^b^	110.57 ± 0.25^a^	117.01 ± 5.12^a^
**Bitter flavor**				
Leu	48.67 ± 0.25^c^	23.08 ± 0.75^d^	120.87 ± 1.04^b^	154.43 ± 4.27^a^
Met	30.03 ± 1.26^c^	24.35 ± 1.58^d^	57.84 ± 1.35^b^	71.93 ± 2.14^a^
Val	117.13 ± 1.05^c^	43.21 ± 0.85^d^	162.68 ± 3.12^b^	181.23 ± 3.16^a^
Ile	23.02 ± 0.45^c^	15.06 ± 0.55^d^	65.27 ± 1.32^b^	92.12 ± 4.12^a^
**Acid flavor**				
Phe	57.98 ± 1.46^c^	39.11 ± 1.47^d^	86.52 ± 0.62^b^	136.27 ± 2.81^a^
Asp	10.05 ± 0.11^b^	11.96 ± 0.19^b^	42.65 ± 1.07^a^	11.81 ± 0.15^b^
His	72.56 ± 1.49^c^	57.36 ± 0.25^d^	143.97 ± 1.12^b^	310.44 ± 5.75^a^
Glu	151.85 ± 1.05^c^	98.37 ± 0.65^d^	433.13 ± 2.11^a^	320.19 ± 4.25^b^
**Aged flavor**				
Tyr	22.82 ± 2.15^c^	17.21 ± 1.05^d^	79.48 ± 1.21^b^	94.11 ± 0.61^a^
Lys	107.28 ± 0.37^c^	39.34 ± 0.14^d^	383.32 ± 1.15^a^	285.26 ± 5.76^b^
Cys	26.26 ± 0.12^b^	11.29 ± 0.74^c^	45.08 ± 1.27^a^	37.15 ± 1.85^b^
Arg	14.31 ± 0.18^d^	25.27 ± 0.52^c^	153.38 ± 0.95^b^	177.05 ± 2.83^a^
TFAAs	1126.32 ± 21.75^c^	768.77 ± 18.27^d^	2699.15 ± 39.45^b^	2891.57 ± 39.71^a^

Data are expressed as mean ± standard deviation (n = 3).

Different superscripted letter in the same line means significant differences (*P* < 0.05).

PTW, plasma treated water.

### Volatile compound analysis

**Figure 4:**
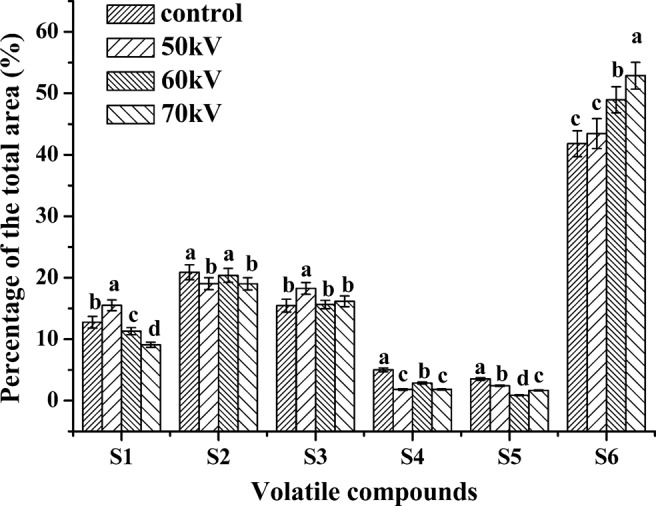
Different kinds of volatile compounds (S1, Alcohols; S2, Aldehydes; S3, Ketones; S4, Esters; S5, Acids; S6, Hydrocarbons) in the dried pork loins cured with PTW brine and the untreated brine.

In total, 54 volatile compounds were found in four groups of Chinese dried pork loin by GC/MS (Table 4). The volatile compounds were classified as ten aldehydes, five esters, five alcohols, six ketones, one acid, and twenty-seven hydrocarbons. Figure 4 shows that PTW used as a curing process could significantly (*P* < 0.05) affect the formation of volatile compounds in dried pork loin. To date, there is limited literature on the flavor of dried pork loin manufactured by PTW brine. It is well known that most of the volatile compounds generated from lipid oxidation and protein degradation, in addition Strecker degradation and the Maillard reaction contributed to the formation of flavor^41^. In this study, 3-methyl-butanol, 1-hexanol and 1-heptanol were only detected in the PTW treatment. 1-Octen-3-ol was the most abundant alcohol and is an unsaturated alcohol produced by linoleic acid β-oxidation that contributes a mushroom flavor^34^. Garcia-Gonzalez *et al*.^42^ found that 1-octen-3-ol contributed to the flavor of cured meat products since it has a low threshold value. In this study, lipid oxidation was prevented by PTW treatment, causing the decline of 1-octen-3-ol compared with that in the untreated control. 3-Methyl-butanol also had a low threshold, which came from the reduction of 3-methyl-butanal and the Strecker reaction^41^.

**Table 4:** Volatile compounds detected in the dried pork loin (percentage of the total peak area) cured with PTW brine and the untreated brine.

Category	Volatile compounds	Control	50 kV	60 kV	70 kV
Alcohols	3-methyl-Butanol	\	3.64 ± 0.11^a^	2.05 ± 0.07^b^	1.05 ± 0.06^c^
	1-Hexanol	\	2.35 ± 0.11^a^	2.58 ± 0.05^a^	2.61 ± 0.07^a^
	1-Heptanol	\	3.26 ± 0.21^a^	1.21 ± 0.05^b^	0.93 ± 0.03^c^
	1-Octen-3-ol	7.58 ± 0.42^a^	6.26 ± 0.15^b^	5.46 ± 0.31^c^	3.60 ± 0.05^d^
	1-Pentanol	5.18 ± 0.35^a^	\	\	0.91 ± 0.05^b^
Aldehydes	Nonanal	9.13 ± 0.35^a^	6.81 ± 0.31^b^	5.03 ± 0.24^c^	5.55 ± 0.15^c^
	Hexanal	\	2.08 ± 0.05^c^	5.58 ± 0.45^a^	3.51 ± 0.21^b^
	Heptanal	0.91 ± 0.03^a^	0.99 ± 0.03^a^	0.87 ± 0.05^b^	0.76 ± 0.03^c^
	Decanal	0.20 ± 0.02^b^	0.39 ± 0.05^a^	\	\
	3-methy-Butanal	3.82 ± 0.25^c^	2.94 ± 0.75^d^	4.85 ± 0.72^b^	6.17 ± 0.86^a^
	Benzaldehyde	3.19 ± 0.75^a^	2.19 ± 0.15^b^	1.60 ± 0.25^c^	1.57 ± 0.21^c^
	(Z)-2-Heptenal	\	1.55 ± 0.15^a^	0.76 ± 0.05^b^	0.48 ± 0.02^c^
	(E)-2-Octenal	\	1.49 ± 0.23^a^	0.78 ± 0.03^b^	0.54 ± 0.11^c^
	3-hydroxy-Butanal	3.63 ± 0.18	\	\	\
	Benzeneacetaldehyde	\	0.59 ± 0.05^b^	0.91 ± 0.03^a^	0.42 ± 0.02^c^
Ketones	2,3-Octanedione	\	9.65 ± 0.78^a^	6.20 ± 0.45^c^	8.01 ± 0.65^b^
	2,3-hydroxy-Butanone	\	7.98 ± 1.05^a^	6.83 ± 0.72^b^	8.15 ± 0.55^a^
	2-Heptanone	5.09 ± 0.25^b^	\	1.99 ± 0.07^a^	\
	2-Nonanone	3.54 ± 0.28	\	\	\
	2,3,3-trimethyl-Cyclobutanone	4.07 ± 0.55	\	\	\
	3-Octen-2-one	2.76 ± 0.16^a^	0.64 ± 0.03^b^	0.62 ± 0.04^b^	\
Esters	Oxalic acid, isobutyl nonyl ester	\	0.58 ± 0.05^a^	0.34 ± 0.05^b^	0.28 ± 0.03^b^
	Oxalic acid, bis(2-ethylhexyl) ester	2.53 ± 0.05^a^	0.60 ± 0.02^d^	1.42 ± 0.15^b^	0.90 ± 0.07^c^
	Oxalic acid, 2-ethylhexyl isohexyl ester	1.24 ± 0.21^a^	0.33 ± 0.03^c^	0.65 ± 0.04^b^	0.34 ± 0.05^c^
	n-Butyric acid, 2-ethylhexyl ester	1.25 ± 0.15^a^	0.31 ± 0.03^c^	0.46 ± 0.02^b^	0.56 ± 0.04^b^
	Sulfurous acid, butyl decyl ester	\	\	\	0.54 ± 0.02
Acids	3-methyl-Butanoic acid	3.53 ± 0.45^a^	2.42 ± 0.27^b^	0.87 ± 0.04^d^	1.66 ± 0.12^c^
Hydrocarbons	D-limonene	1.71 ± 0.54^a^	0.60 ± 0.07^b^	0.54 ± 0.09^b^	0.43 ± 0.08^bc^
	p-xylene	1.86 ± 0.07^a^	0.21 ± 0.02^c^	0.23 ± 0.04^c^	0.39 ± 0.07^b^
	o-xylene	1.51 ± 0.07^a^	0.45 ± 0.04^b^	0.29 ± 0.01^c^	0.18 ± 0.03^c^
	1,2,4-trimethyl-Benzene	2.52 ± 0.21^a^	1.16 ± 0.04^b^	1.15 ± 0.09^b^	1.04 ± 0.11^b^
	2-pentyl-Furan	8.63 ± 0.65	\	\	\
	2,6-dimethyl-Pyrazine	1.90 ± 0.02^a^	1.10 ± 0.07^b^	1.66 ± 0.25^a^	0.84 ± 0.05^c^
	Tetramethyl-Pyrazine	1.88 ± 0.55^b^	2.11 ± 0.15^b^	3.81 ± 0.41^a^	4.07 ± 0.34^a^
	2,4,4-Trimethyl-1-hexene	\	\	\	0.54 ± 0.02
	Pentane, 2,3,3-trimethyl-	\	3.15 ± 0.22	\	\
	Hexane, 3-ethyl-4-methyl-	\	0.57 ± 0.02^b^	0.66 ± 0.03^b^	2.77 ± 0.21^a^
	Heptane, 2,2,4,6,6-pentamethyl-	\	12.26 ± 0.72^c^	16.34 ± 0.55^b^	17.34 ± 0.85^a^
	Heptane, 2,4-dimethyl-	\	\	2.54 ± 0.24^a^	2.68 ± 0.37^a^
	Octane	13.75 ± 0.95	\	\	\
	Octane, 3,5-dimethyl-	\	1.63 ± 0.21^c^	2.76 ± 0.31^a^	2.40 ± 0.26^b^
	Octane, 4,5-dipropyl-	\	\	\	0.63 ± 0.29
	Nonane, 4,5-dimethyl-	\	0.38 ± 0.05^b^	\	1.80 ± 0.21^a^
	Decane	\	\	\	2.83 ± 0.75
	Decane, 3,8-dimethyl-	\	6.73 ± 1.25^a^	2.37 ± 0.11^c^	3.46 ± 0.22^b^
	Decane, 2,5,6-trimethyl-	\	\	\	1.04 ± 0.23
	Undecane, 3,6-methyl-	\	5.68 ± 0.35^b^	9.07 ± 0.82^a^	1.72 ± 0.23^c^
	Undecane	\	\	1.84 ± 0.12^a^	1.14 ± 0.20^b^
	Dodecane	2.49 ± 0.25^a^	1.41 ± 0.04^b^	1.58 ± 0.05^b^	2.59 ± 0.34^a^
	Dodecane, 2,6,10-trimethyl-	\	1.01 ± 0.05^a^	0.63 ± 0.01^b^	1.20 ± 0.02^a^
	Tridecane	2.82 ± 0.21^a^	1.68 ± 0.05^b^	1.47 ± 0.04^b^	0.65 ± 0.03^c^
	1H-Indole, 5-methyl-2-phenyl-	\	1.49 ± 0.09^a^	\	0.24 ± 0.02^b^
	2-Ethyl-acridine	3.21 ± 0.64	\	\	\
	Butane, 1-methoxy-3-methyl-	\	1.42 ± 0.06^b^	1.14 ± 0.06^c^	2.23 ± 0.14^a^

Data are expressed as mean ± standard deviation (n = 3).

Different superscripted letter in the same line means significant differences (*P* < 0.05). ‘\’ = not detected.

Aldehydes have an important impact on the overall flavor of cured meat products due to their low perception threshold^43^. Among the straight chain aldehydes detected in all four treatments, nonanal was the most abundant compound, except in the 60 kV treatment, which showed a significantly higher (*P* < 0.05) nonanal concentration in the control than that in the PTW treatments. This result was consistent with the TBARS results since nonanal is generally considered a product of lipid oxidation^44^. However, we found that the results of hexanal were in contradiction with the TBARS values, which could be attributed to the difference in sources of hexanal and MDA and various rates of generation; in addition, further reactions with other substances between hexanal and MDA could cause this result as well^21^. The significantly lower (*P* < 0.05) levels of heptanal and decanal in the 60 kV and 70 kV treatments than those in the other two treatments are in agreement with a lower level of lipid oxidation in the 60 kV and 70 kV treatments.

The ripening temperature used in this study was shown to be high enough for initiating the Maillard reaction and Strecker degradation in the meat process^45^. Hence, the lowest amount of 3-methyl-butanal in the 50 kV treatment among that in all treatments could be explained by the lowest content of FAAs being exhibited in the 50 kV treatments since 3-methyl-butanal is related to branched amino acids involved in the Maillard reaction and Strecker degradation^46^. The higher benzaldehyde content in the control compared with that in the PTW treatments was related to the higher TBARS values in the control since benzaldehyde is produced by the decomposition of α-linolenic acid^47^.

2,3-Octanedione and 2,3-hydroxy-butanone formed from carbohydrate fermentation^46^ were only found in the PTW treatments. 2-Heptanone and 2-nonanone are the products of lipid oxidation with ethereal, butter, or spicy flavors^43^, which were significantly higher in the control. The esters could be generated by the esterification reactions between acids and alcohols^47^. Two more ester compounds were found in PTW-processed dried pork loin, which may be due to the increased amount of alcohols in the PTW-treated products. Only one acid was detected, which may be related to the shorter processing time compared to that of other meat products. 3-Methyl-butanoic acid with a unique cheese flavor could be formed by the oxidation of 3-methyl-butanal^48^. Due to the high threshold value of straight chain hydrocarbons, even the high content of straight chain hydrocarbons had no significant impact on the flavor of cured meat products^41^. Aromatic hydrocarbons, such as d-limonene, came from the animal diet, while p-xylene, o-xylene, and 1,2,4-trimethylbenzene are benzene derivatives^47^. 2-Pentylfuran derived from linoleic acid and other n-6 fatty acids were not found in the PTW-treated products. Pyrazine compounds belonging to nitrogen-containing heterocyclic compounds with a roasted aroma were found in all treatments. Kim, *et al*.^3^ had demonstrated that there were no significant sensory property differences between cooked pork loins treated with DBD-CP and the untreated control.

## Conclusion

In summary, we prepared Chinese dried pork loin using PTW brine as a novel curing process. The results showed that compared with the traditional method, dried pork loins made by treatment with PTW had no undesirable properties according to the analysis of physicochemical properties and volatile compounds. Thus, we concluded that PTW brine could be a potentially safe curing process for meat products. The physicochemical properties of the PTW - treated and untreated products in this study were mainly concentrated on regarding the impacts on color, lipid oxidation and FAA of PTW. The significantly higher a* value in the 70 kV treatment than those in the other treatments provided the characteristic cured color to the meat products, which is important for consumers. According to our DBD-CP system, the 70 kV treatment could result in significant increases in the accumulation of FAA, which has a great influence on the specific taste. Moreover, the TBARS values in the 70 kV treatment were markedly lower than those in the control, suggesting that the 70 kV treatment could increase the oxidative stability of meat products. Hence, based on these analyses, it is recommended to use 70 kV in the treatment of PTW for curing meat products. However, even though PTW is already proven to be a purified water without chemical additives, the safety of meat products processed with PTW warrants further research to satisfy authorities and consumers.
